# Study protocol of comprehensive risk evaluation for anorexia nervosa in twins (CREAT): a study of discordant monozygotic twins with anorexia nervosa

**DOI:** 10.1186/s12888-020-02903-7

**Published:** 2020-10-14

**Authors:** Maria Seidel, Stefan Ehrlich, Lauren Breithaupt, Elisabeth Welch, Camilla Wiklund, Christopher Hübel, Laura M. Thornton, Androula Savva, Bengt T. Fundin, Jessica Pege, Annelie Billger, Afrouz Abbaspour, Martin Schaefer, Ilka Boehm, Johan Zvrskovec, Emilie Vangsgaard Rosager, Katharina Collin Hasselbalch, Virpi Leppä, Magnus Sjögren, Ricard Nergårdh, Jamie D. Feusner, Ata Ghaderi, Cynthia M. Bulik

**Affiliations:** 1 Division of Psychological and Social Medicine and Developmental Neurosciences, Faculty of Medicine, Technische Universität Dresden, Fetscherstraße 74, 01307 Dresden, Germany; 2 Department of Medical Epidemiology and Biostatistics, Karolinska Institutetet, Nobels väg 12A, 17165 Stockholm, Solna Sweden; 3 Department of Psychiatry, Harvard Medical School, Boston, MA USA; 4 Eating Disorders Clinical and Research Program, Massachusetts General Hospital, Boston, MA USA; 5 Centre for Psychiatry Research, Department of Clinical Neuroscience, Karolinska Institutet, Stockholm, Sweden; 6 Stockholm Health Care Services, Region Stockholm, Stockholm Centre for Eating Disorders, Stockholm, Sweden; 7 Social, Genetic & Developmental Psychiatry Centre, Institute of Psychiatry, Psychology & Neuroscience, King’s College London, London, UK; 8 UK National Institute for Health Research (NIHR) Biomedical Research Centre (BRC), South London and Maudsley NHS Foundation Trust, London, UK; 9 National Centre for Register-based Research, Aarhus Business and Social Sciences, Aarhus University, Aarhus, Denmark; 10 Department of Clinical Neuroscience, Karolinska Institutet, Stockholm, Sweden; 11 Department of Clinical Medicine, University of Copenhagen, Copenhagen, Denmark; 12 Eating Disorder Research Unit, Mental Health Center Ballerup, Ballerup, Denmark; 13 Department of Women’s and Children’s Health, Karolinska Institutet, Stockholm, Sweden; 14 Department of Psychiatry and Biobehavioral Sciences, University of California Los Angeles, Los Angeles, CA USA; 15 Department of Psychiatry, University of North Carolina at Chapel Hill, Chapel Hill, NC USA; 16 Department of Nutrition, University of North Carolina at Chapel Hill, Chapel Hill, NC USA

**Keywords:** Twin study, Risk factors, Study protocol, Cognitive functions, Neuroimaging, Genetics, Metabolism, Microbiota, Anorexia nervosa

## Abstract

**Background:**

Anorexia nervosa (AN) is a severe disorder, for which genetic evidence suggests psychiatric as well as metabolic origins. AN has high somatic and psychiatric comorbidities, broad impact on quality of life, and elevated mortality. Risk factor studies of AN have focused on differences between acutely ill and recovered individuals. Such comparisons often yield ambiguous conclusions, as alterations could reflect different effects depending on the comparison. Whereas differences found in acutely ill patients could reflect state effects that are due to acute starvation or acute disease-specific factors, they could also reflect underlying traits. Observations in recovered individuals could reflect either an underlying trait or a “scar” due to lasting effects of sustained undernutrition and illness. The co-twin control design (i.e., monozygotic [MZ] twins who are discordant for AN and MZ concordant control twin pairs) affords at least partial disambiguation of these effects.

**Methods:**

Comprehensive Risk Evaluation for Anorexia nervosa in Twins (CREAT) will be the largest and most comprehensive investigation of twins who are discordant for AN to date. CREAT utilizes a co-twin control design that includes endocrinological, neurocognitive, neuroimaging, genomic, and multi-omic approaches coupled with an experimental component that explores the impact of an overnight fast on most measured parameters.

**Discussion:**

The multimodal longitudinal twin assessment of the CREAT study will help to disambiguate state, trait, and “scar” effects, and thereby enable a deeper understanding of the contribution of genetics, epigenetics, cognitive functions, brain structure and function, metabolism, endocrinology, microbiology, and immunology to the etiology and maintenance of AN.

## Background

We present the study protocol for the Comprehensive Risk Evaluation for Anorexia nervosa in Twins (CREAT): A study of monozygotic (MZ) twins who are discordant for anorexia nervosa (AN). AN is a complex and serious disorder characterized by prolonged undernutrition, low weight, weight/shape concerns [3] and is often accompanied by pathologically elevated physical activity. Individuals with AN present with considerable somatic and psychiatric comorbidity [130]. Mortality associated with AN is significantly elevated [2] and higher than in other serious psychiatric disorders, such as schizophrenia, bipolar disorder, and major depression [21]. Rates of relapse and hospital readmission are high [34, 116, 130]. Consequently, the disorder places a major social and financial burden on patients, family members, and the health care system [112]. No medications are effective in the treatment of the core symptoms of AN [130] and drug development has been hindered by a lack of understanding of the (neuro) biology of the disorder and a systematic integration of those biological factors with environmental components [88]. Until we gain a deeper understanding of the biology of the illness, we will be unable to determine which factors place individuals, or more nuanced questions such as which factors enable individuals with AN who do not die from the disorder to maintain dangerously low body mass indices (BMI), often for prolonged periods of time, without succumbing to medical complications of severe starvation or other opportunistic illnesses [16].

### Study overview

To address these aforementioned knowledge gaps and to inform treatment development, we employ a co-twin control design—an investigation of discordant MZ twins (i.e., twin pairs in which one twin has a target disorder and the other remains unaffected, despite being past the age at risk of onset for the illness). We are recruiting ~ 50 pairs of female MZ twins in which one has or has had AN, and ~ 50 pairs of age matched female control MZ twins in which neither twin has any history of eating disorders symptoms.

The study is made possible by the existence of the Swedish [69, 75] and Danish [107, 108] twin registers and the rich national health registers in both countries [29, 72, 73, 84]. This will allow us to identify all twins in each country in which one twin has an identified diagnosis of AN.

As discordant MZ twins are both rare and highly valuable to health research, we have assembled an experienced interdisciplinary team to design a comprehensive investigation that will thoroughly explicate the role of genetic, epigenetic, neurocognitive, neural, endocrinological, metabolic, microbial, immunological, and environmental factors associated with AN. We employ a longitudinal, multimodal co-twin control design that includes an experimental component, namely an 18-h overnight fast. This experimental component affords us the opportunity to observe differential response to fasting across a range of biological and psychological outcome domains.

### The value of co-twin control designs

The study of discordant MZ twins represents a powerful research strategy to explore factors that influence differences between individuals who are fundamentally genetically identical. Discordant MZ twins are a natural experiment in which appropriate matching occurs. The validity of twin research also rests on the assumption that environmental factors are also shared equally between MZ twins (during early life). As one can therefore assume background genetic matching, observed differences in the relevant traits can be due to differential exposures, consequences of the illness, epigenetic processes, or the state of recovery of the affected twin. Due to the prevalence of AN, co-twin control studies in this field have been rare and small [54, 93, 114, 119]. Below we describe each component of CREAT including a concise background, relevance, and expected outcomes.

### Study aims and protocol overview

**Fig. 1:**
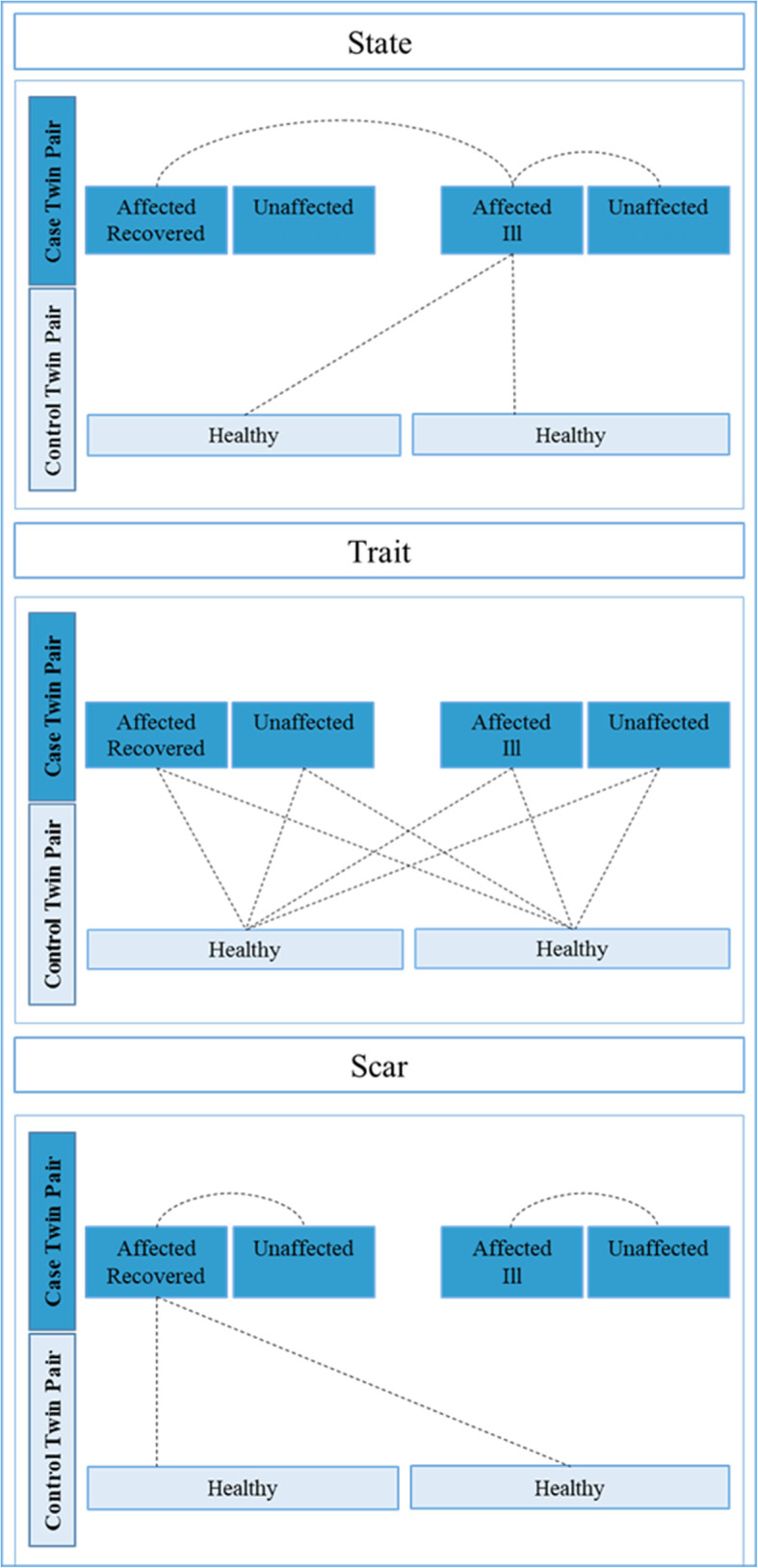
Overview of possible comparisons between twins to investigate whether effects constitute state, trait, or “scar” markers. Lines indicate comparisons where observed differences would imply state, trait, or “scar” effects respectively

**Fig. 2:**
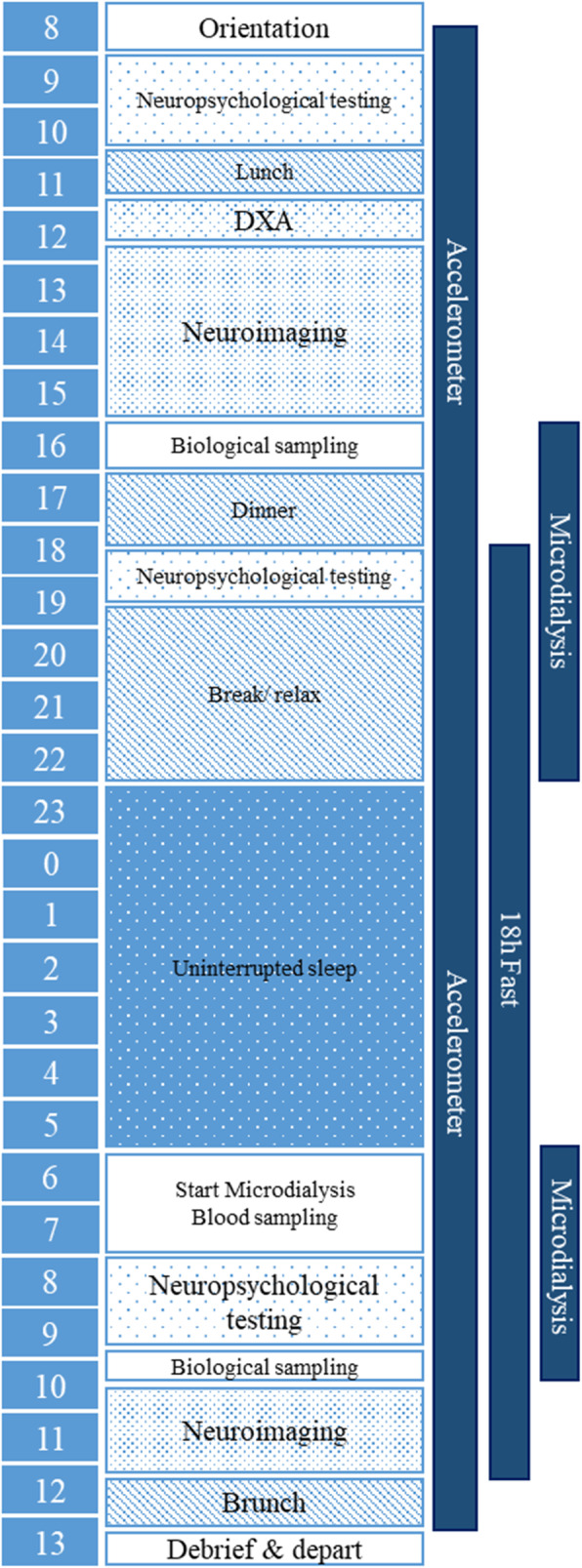
Overview of the study schedule. Participants undergo a 30 h protocol, starting at 8:30 am in the morning of day 1 until approximately 1:00 pm of day 2. Study components flow as per Fig. 3. Individuals wear an accelerometer for 29 h of the study. The fasting period commences at 18:30 following dinner on day 1 and continues until 12:30 on day 2. Neurocognitive testing, neuroimaging, biological sampling, and microdialysis are conducted before (T1) and after (T2) the experimental fast

Previous studies on risk factors for AN have often focused on differences between acutely ill and recovered individuals. However, this design lends itself to some ambiguity in interpretation. Differences observed in acutely ill patients could reflect state effects due to acute starvation or acute disease-specific factors, or could represent underlying traits. Alternatively, observations in recovered individuals could reflect either an underlying trait or a “scar”, due to lasting effects of sustained undernutrition and illness [89]. We anticipate that approximately half of the participating twins with AN will be recovered. Recovery criteria in the current study follow a modified version of Bardone-Cone et al. [6] and include 1) physical recovery: BMI ≥ 18.5 kg/m2; 2) behavioral recovery: no binge eating, purging, or fasting in the past year, and 3) psychological recovery: all Eating Disorder Examination-Questionnaire (EDE-Q; [30]) subscales within 1 SD of age-matched community norms. Further, we anticipate that about 25% will be actively ill (i.e., meet threshold diagnostic criteria), and 25% will be partially recovered, falling between ill and recovered on either physical, behavioral, or psychological parameters. Using a discordant MZ twin design as well as different recovery states the current study will help to disentangle not only state and trait effects, but also “scar” effects (comparisons as outlined in Fig. 1) across several different investigational domains. Briefly, in a two-day study, all participating twins undergo blood sampling, neuroimaging, neurocognitive testing, body composition measurement via dual X-ray absorptiometry (DXA), accelerometer-based activity monitoring, microdialysis, self-report questionnaires, and structured interviews over a 28–30 h period with standardized food intake and a monitored overnight fast (Fig. 2).

### Experimental overnight fast

Appetite regulation and, therefore, the motivation to eat reflects a complex integration of several physiological signals influencing hunger, satiety, physical activity, and cognitive processes that affect the subjective value of food [60, 120]. The relentless and pervasive caloric restriction during periods of extreme low body weight and hallmark endocrine disturbances that accompany eating behavior [101] suggest disrupted homeostatic influences on reward processing [33, 85]. The majority of studies in acute AN to date are unable to disaggregate the effects of fasting from confounding effects of long-term mal- or undernutrition. However, altered responses to short-term fasting persist even after weight restoration [44, 124], suggesting persistent traits underlying the AN phenotype and associated pathological eating behavior. Our experimental overnight fast allows us to document differences between affected twins and their unaffected co-twins, as well as between discordant affected MZ case twins and concordant unaffected MZ control twins in neuronal, neurocognitive, metabolic, endocrinological, and psychological response to short-term food restriction by repeating assessments across a number of domains pre- and post-fast while holding other variables constant.

### Genomics & Epigenetics

AN is highly familial. Twin studies estimate between 28 and 74% of the variance in liability is attributable to genetic factors [127]. The most recent genome-wide association study (GWAS) identified eight genomic loci associated with AN, with genetic correlations suggesting both metabolic and psychiatric risk factors for the illness. These recent findings advance previous notions of a re-conceptualization of the illness as a metabo-psychiatric condition [42, 58, 121]. In addition to genetic risk, the AN phenotype and disease course could also be shaped by epigenetic mechanisms. Epigenetics refers broadly to the regulation of gene expression without a change in the DNA coding sequence. Unlike the genome sequence, epigenetic marks can be subject to environmental stimuli starting immediately after conception [79], which play a role in AN, including hormones, nutrition, lifestyle, and intestinal microbiota [2, 79, 91]. Epigenetic studies to date have focused solely on the role of DNA methylation [45, 46]. We expect that affected twins will differ in their epigenetic signature, including DNA modifications, histone modifications, and non-coding RNAs, and that differences between discordant twins, especially the recovery status of the affected twin, may aid identifying illness-related (state) effects (Fig. 1 A) versus “scar” effects from chronic undernutrition (see Fig. 1 C).

### Neuropsychology

Several neurocognitive functions such as set-shifting, value-based decision making, and central coherence have been reported to be altered in AN [1, 115, 123]. The extent to which these alterations are trait or state markers remains unclear. Observations in individuals with acute AN, recovered AN, and unaffected sisters or mothers suggest that deficiencies in set shifting may be a trait marker in AN [61, 63, 71, 97, 123]. On the other hand studies in adolescent patients have not replicated impairments in set-shifting, suggesting that chronic illness might have contributed to positive findings [62].

Consistent with the clinical presentation of elevated self-control in AN [53], delay discounting (i.e., the depreciation of the value of a reward related to the time of retrieval), and probability discounting have been reported to be altered in AN patients [8, 26, 110, 111]. Some studies suggest that AN patients have a preference for larger delayed rewards when compared to healthy individuals whereas studies in younger AN patients did not find differences in discounting behavior [56, 96]. Multiple studies have also observed weak central coherence - reduced global and integrated processing and enhanced focus on detail [35] - in underweight individuals with AN, and in some studies in unaffected relatives [37, 61, 63, 98], yet there are conflicting reports about whether this persists in weight-restored or recovered states [61, 63, 122]. Preliminary evidence in healthy individuals suggests that short term fasting affects both set-shifting and central coherence [7, 13, 87]. The experimental overnight fast allows us to observe whether short term food deprivation differentially modifies cognitive functioning in affected versus unaffected twins, and in comparison to concordant unaffected MZ control twins.

### Neuroimaging

Structural brain changes, i.e., the reduction of gray matter (GM) and white matter (WM), are most pronounced in acutely underweight patients with AN [55, 57, 104] with normalization occurring quite rapidly after short-term recovery [9, 10, 118]. Standard designs have been unable to differentiate state (due to undernutrition, even after recovery) and trait effects. The co-twin control design has the potential to aid in disaggregating these effects and potentially identifying trait effects in structural changes in AN.

Accumulating evidence of functional MRI (fMRI) data further points to alterations within established resting state networks [11, 12, 24, 77, 100] as well as during specific tasks. Domains reported to be altered in AN include reward processing [85], food-cue reactivity [129], emotion recognition and processing [95, 103, 129], and general visual processing [66, 67]. The relative contribution of trait, state, or “scar” factors of these alterations will be visible in the difference between a) the affected and currently ill and the unaffected discordant twin (state), b) the unaffected discordant twin and twin controls (trait), and c) the affected now recovered and unaffected discordant twin (“scar”) respectively (see Fig. 1). Reward processing and food cue reactivity are expected to be modulated by the nutritional status of the patients, i.e., whether patients are acutely ill or weight-restored and whether patients are scanned after a period of fasting or after a meal [64, 124].

### Endocrinology & Metabolism

AN has far reaching effects on most endocrine systems [47, 101]. This is unsurprising as the endocrine system plays a major role in adaptation to an ever-changing environment with varying demands on how to eat, use, and store energy. Being able to adapt to periods of food restriction, negative energy balance, and even starvation is essential for most animals and, not surprisingly, starvation has broad impact on metabolism, reproduction, and behavior. In the CREAT study we will compare endocrine and metabolic responses to food restriction between discordant MZ twins. Early metabolic changes in response to the overnight fast will be captured by microdialysis. The microdialysis technique, described in detail elsewhere [52], places a probe in subcutaneous fat, allowing observation of how glucose homeostasis is maintained via different metabolic pathways, e.g., activation of lipolysis and gluconeogenesis. We will also measure key metabolic hormones such as cortisol, ghrelin, and thyroid hormones. Our experimental design will enable us to differentiate between early differences in response to fasting from secondary effects of starvation as well as separate biomarkers of negative energy balance from biomarkers of the disorder. We hypothesize that an identifiable metabolic/endocrine response to negative energy balance is associated with an increased risk of developing AN.

### Intestinal microbiome

The human intestinal microbiota is a complex and dynamic community of microorganisms that plays a fundamental role in many host processes including energy metabolism [18] and immunity [36], as well as broad regulation of mood [27] and behavior [83]. Dysbiosis of the intestinal microbiota has been associated with a diverse range of health deficits [48]. Several studies have confirmed differences in the diversity and composition of the intestinal microbiota in individuals with AN and controls, and across the course of therapeutic renourishment [4, 14, 59, 74, 81, 82, 102]. It is possible that alterations in the intestinal microbiota are adaptive responses to an extreme intestinal environment during acute stages of AN, allowing survival during periods of starvation. Another possibility is that the gut microbiota plays a co-causal role in AN, although the mechanism for this requires further explication. Although the aforementioned studies provide valuable findings that give us an outlook for future investigations, their study designs do not allow elaborating beyond associations nor do they disambiguate state, trait, or “scar” effects. We will compare fecal microbiota (as a proxy to study intestinal microbiota) in affected versus discordant unaffected co-twins (to identify state-related alterations), unaffected discordant twins and twin controls (to pinpoint trait-related changes), and affected recovered twins versus unaffected discordant co-twins (to assess “scar”-related alterations). Relating the microbiota data to dietary and metabolic data will help explicate the role of microbial variability in the availability and absorption of nutrients from the gut and, potentially, in modulating host factors. As epigenetic regulation is a potential mechanism through which gut microbiota and microbial metabolites can affect host factors, we will also investigate the associations between epigenetic data and microbiota data in our study groups.

### Immunology

The blood-brain barrier and the blood-cerebrospinal fluid barrier separate the neuroimmune system from the peripheral immune system. However, the communication between them is possible via cytokines that can influence a variety of brain functions relevant to behaviour including neurotransmitter metabolism, neuroendocrine function, synaptic plasticity, and neurocircuits that regulate mood, anxiety, and motivation [19]. Higher concentrations of the cytokines tumor necrosis factor α (TNFα) and interleukin 6 (IL-6) have been reported in AN compared with healthy controls but may not be specific for AN [25]. Longitudinal investigations of cytokines have not yet been conducted, therefore, it is difficult to judge whether these alterations are trait-related or a sequelae of AN [25], but initial evidence suggests that individuals with AN seem to carry genetic variants predisposing them to lower C-reactive protein (CRP) concentrations [70]. Additionally, a bidirectional relationship between AN and autoimmune diseases, such as ulcerative colitis and Crohn’s disease, and an increased risk for AN after a preceding diagnosis of type 1 diabetes has been reported and replicated [43, 92, 126, 128]. The co-occurrence is not yet fully understood, but no shared genetic liability between autoimmune diseases and AN has been detected so far [117]. The existing findings encourage the exploration of other factors, such as infections, medication [15], insulin, diet or starvation effects as potential candidates to explain the relationship between AN and abnormalities of the immune system and/or autoimmune diseases. We also aim to understand whether the effects of either ongoing subtle cell damage or an unusual pattern of inflammation are exacerbated by a lack of adipose tissue which alters the expected production of mediators and the interrelationship of cytokines.

## Methods

### Basic characteristics and diagnosis

#### Participants

We are recruiting female MZ twins rigorously discordant for AN and healthy non-dieting age- matched female concordant unaffected MZ twin controls. The twins are identified and recruited from the Swedish Twin Registry (STR) [69] and the Danish Twin Registry (DTR) [107, 108]. The STR was first established in the late 1950s and includes about 200,000 twins, providing an invaluable and unique resource for scientific studies such as CREAT. The Danish Twin Register (DTR) was initiated in 1954 and covers all twin cohorts established in Denmark since 1870. The DTR contains more than 85,000 twin pairs and a similarly valuable resource for health research.

We intend to enroll 50 MZ discordant twin pairs and 50 MZ concordant unaffected control pairs (N_total_ = 200). Participants are reimbursed for participation with a fixed amount of 4000 Swedish kronor, which they receive after completion of the study.

#### Inclusion-exclusion criteria

**Fig. 3:**
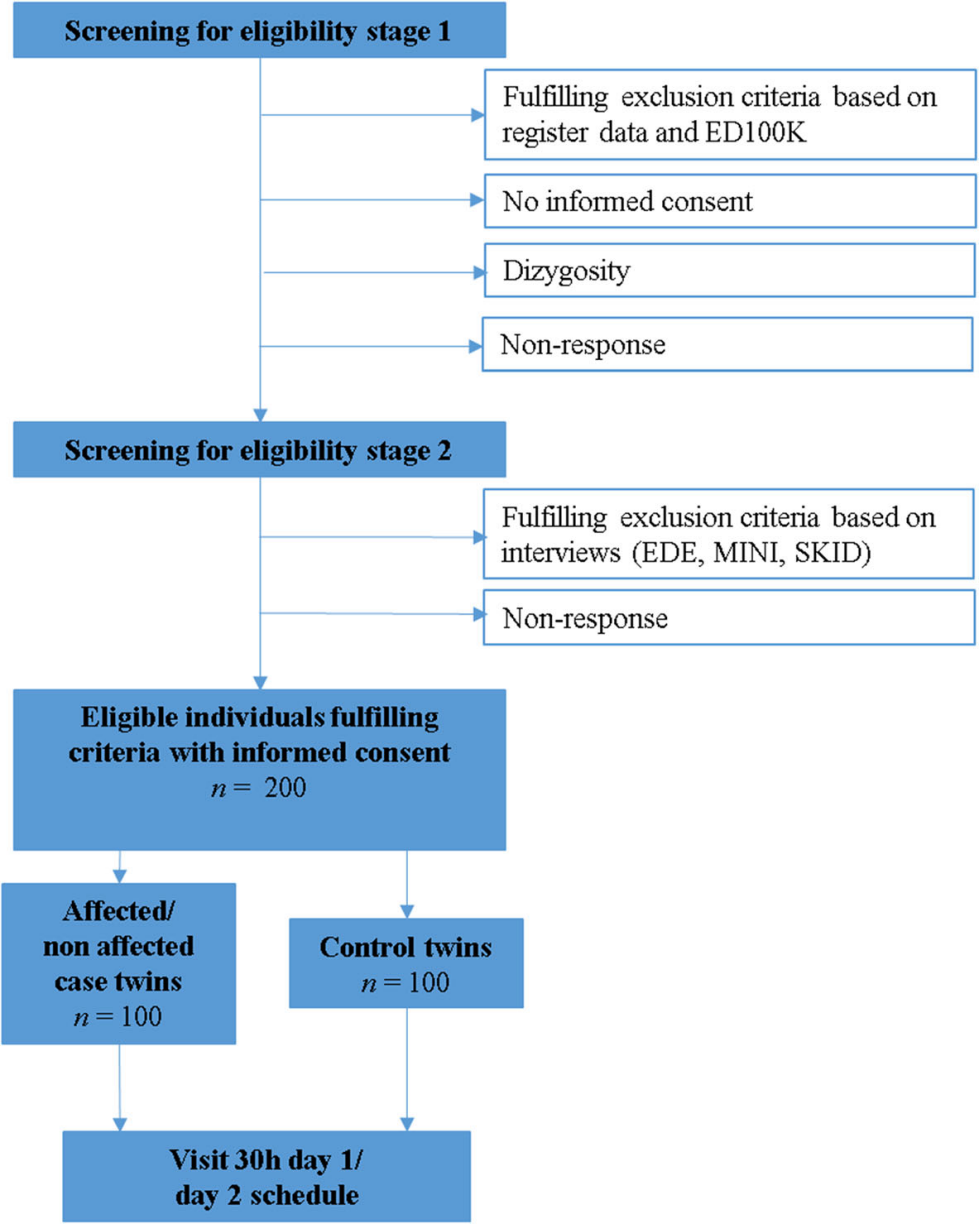
Inclusion (↓)/Exclusion (→) procedures for recruitment. Pre-screening begins at Stage 1. Individuals are screened with the ED100K online questionnaire [113] for lifetime eating disorder symptoms as well as genetic testing. Participants are excluded if screening reveals them to be dizygotic or if the unaffected co-twin is discovered to be affected. Screening at Stage 2 is more intensive and includes telephone interviews by trained interviewers conducting the Eating Disorder Examination [EDE [23]], Mini-International-Neuropsychiatric-Interview [MINI, [106]], and the Eating Disorders section of the Structured Clinical Interview for DSM-5 [SCID-5 [32]]. If all criteria fulfilled and participants remain interested in participating, they are invited to participate in the 30 h study protocol

Stage 1 Initial screen: The Stage 1 review determines preliminary eligibility (females only, aged between 18 and 50 Fig. 3). Twins who appear to be MZ and discordant for AN based on diagnostic information obtained via participation in the STR [68] are sent an invitation to participate in Stage 1. Danish twins are identified by selecting a cohort of all MZ female twins, age 18–50 from the DTR (*n* = 1617 pairs, where both twins are alive per 1st of October 2018) or via social media or contacts with healthcare providers. Via the Danish National Patient Register [73] and Psychiatric Central Research Register [84], this cohort will be matched for the diagnosis of AN and contact information obtained via the Civil Registration System. After giving informed consent, each twin pair is genotyped to verify monozygosity (for details see Supplementary Material 1.1, Additional file 1). In addition to the saliva kits for the monozygosity screening, participants are also provided with a link to a validated diagnostic online questionnaire, ED100K [113] which captures lifetime DSM-5 eating disorder diagnoses. This is a second stage of verifying that affected twins did indeed have AN and that co-twins and control twins are unaffected.

Stage 2 Pre-visit evaluations: MZ twins who are confirmed to be MZ via genotyping and who appear to meet eligibility criteria after Stage 1 undergo a phone-based interview including the complete Eating Disorder Examination (EDE [23]) for assessing current diagnosis of eating disorder, and eating disorder-specific psychopathology. For control twins, a short version of the EDE (only diagnostic questions) is used. The EDE is a reliable, valid, and widely used instrument for diagnosing eating disorders, and has been translated into Swedish and Danish. In both twin groups, the Eating Disorders module of the SCID-5 [32] is used to assess the lifetime illness course and recovery status. The assessments are performed by clinically trained research staff who are experienced in psychiatric diagnostic assessments in general and eating disorders in particular.

Our definition of rigorous discordance is that the affected twin should meet or have met modified DSM-5 criteria for AN at some point in their life, and the unaffected twin should not meet any current or past criteria for AN or any other eating disorder. In addition, the unaffected twin should have been unaffected for at least five years after the onset of their co-twin’s AN, have a lifetime minimum adult BMI above the underweight cut off (18.5 kg/m^2^), and not suffer from other metabolic conditions that could influence body weight or appetite.

After evaluating all inclusion and exclusion criteria, all twin pairs undergo a second phone-based interview using the MINI International Neuropsychiatric Interview 7.0 [MINI, [106]]. The MINI is a structured clinical interview for diagnosing psychiatric disorders.

Control twins (unaffected MZ twins) must both meet the same criteria as the unaffected co-twins of the discordant pairs. Additional exclusion criteria for healthy MZ control twin pairs are: history of psychiatric illness such as recurrent major depression, psychosis, bipolar disorder, or generalized anxiety disorder screened by the MINI International Neuropsychiatric Interview (2015), and ongoing psychotropic medication use. All participants are excluded if they report being pregnant (invited to participate at a later date), are breastfeeding (invited to participate at a later date), have a neurological disease, had brain surgery, or have magnetic materials in the body (contraindicating MRI). MZ twins who are rigorously discordant and unaffected MZ control twins who meet all of these criteria are then invited for further participation.

### Materials and measures

#### Questionnaires

Participants will complete a battery of questionnaires developed to capture a range of relevant behavioral, cognitive, personality, and psychological factors hypothesized to be relevant to risk for eating disorders (Table 1, Supplementary Material 1.2, Additional file 1).

**Table 1:** Overview of questionnaire measures used in the sample. For more detailed information of each of the instruments please refer to the Supplementary Material (1.3.1–1.3.20, Additional file 1

Questionnaire	Measure
MiniMeal-Questionnaire	General diet behaviors
Impulsive Behavior Scale (SUPPS-P)	Impulsivity
Active-Questionnaire (Active-Q)	Physical activity
Eating Disorder Examination - Questionnaire (EDE-Q)	Disordered eating behavior
Quality of Life Inventory (QOLI)	Quality of life
Nicotine Dependency Scale	Nicotine dependence
Hormonal, Menstrual and Reproductive History (HMRH)	Reproductive history
Perception of Teasing Scale (POTS)	Weight and competency teasing
Edinburgh Handedness Inventory	Handedness
Behavioral Inhibition/Approach System (BIS/BAS)	Behavioral inhibition and approach tendencies
Multidimensional Perfectionism Scale (MPS)	Perfectionism
Patient Health Questionnaire (PHQ9)	Depression
Generalized Anxiety Disorder (GAD-7)	Severity of anxiety disorder
Autism Spectrum Quotient (AQ-10)	Position on the autism-normality continuum
Obsessive Compulsive Inventory – Revised (OCI-R)	Compulsive behaviour
Big Five Inventory (BFI)	Extraversion, agreeableness, consciousness, neuroticism, openness
Online 24-Hour Recall Log Book	Nutrition intake
Visual Analogue Scale (VAS)	Hunger and thirst
Positive and Negative Affect Schedule (PANAS)	Positive and negative affect
Stool and Medication Questionnaire	Gastrointestinal symptoms and medication intake

#### Anthropometric measures, diet, and physical activity

Central to our investigation is the exploration of differences in anthropometric measures and body composition, diet, and physical activity among unaffected and affected twins and healthy controls. BMI is calculated from measured height (via fixed stadiometer) and weight (via calibrated digital scale). Percent body fat and lean muscle mass are measured by DXA. Physical activity is measured by the Active-Q Questionnaire (see subsection Questionnaires for more detail). Additionally, participants wear GENEActive Original accelerometers (Activinsights Ltd., UK) for a six-day period on their non-dominant wrist prior to the on-site phase of the study and during the entire stay during the study. We sample vertical, anteroposterior, and mediolateral movement at a rate of 40 Hz to maintain accuracy at an informative level.

#### Genetics

Blood DNA samples from all participants will be genotyped on an appropriate chip at the time of study commencement and after standard quality control [22]. The genomic information will be used to estimate the polygenic loading for different phenotypes, including metabolic, behavioral, and psychiatric traits. The polygenic loading will be estimated by polygenic scores [65] or summary data-based best linear unbiased predictors (SBLUPs) [99] and we will investigate differences in the association of these predictors with other outcome measures. Potentially, these polygenic scores will be combined with the classical twin design [80]. For selected samples, we will consider whole genome or exome sequencing to identify de novo alterations.

#### Epigenetics

As monozygotic twins are nearly 100% genetically identical, the majority of genomic effects influencing differences between each twin of a twin pair will be due to epigenetic changes between the twins. To investigate these epigenetic differences between twins, we will conduct epigenome-wide association studies examining differential methylation and histone modifications [79]. We will correct for potential differences in cell type composition between the four groups by appropriate statistical models [51] and take eating disorder-specific confounders, including diet composition, medication, hormone concentrations, and underlying DNA sequence into account [45].

#### Cognitive functions

Participants complete a battery of neurocognitive tests both online on a laptop and by paper and pencil (Table 2). The neurocognitive battery is administered twice using alternate forms, once before (T1) and once after (T2) the 18 h fasting period. For more detailed description of each task, please see Supplementary Material (1.3, Additional file 1).

**Table 2:** Overview of tests of neurocognitive function and body image distortion applied during timepoint 1 (T1) and timepoint 2 (T2). The Set-shifting battery is based on Wolff et al. [125]; the Value-based- decision making battery (VBDM) based on Pooseh et al. [90]. The Somatomap 3D is conducted according to Ralph-Nearman et al. [94]. The Rey Osterrieth Complex Figure Test [78] was used

Task	Measure	T1	T2
Set-shifting	Set-shifting ability (color-shape, category switching, number-letter task)	X	X
Value-based decision making battery	Delay discounting, reward sensitivity, punishment sensitivity	X	X
Rey-Osterrieth Complex Figures Test	Perceptual organization & visual memory	X	X
Somatomap 3D	Body image distortion	X	X
Raven‘s SPM	Cognitive ability	X	

#### Neuroimaging

Participants complete a protocol of structural (T1-weighted (T1W) and diffusion tensor imaging (DTI)) and functional neuroimaging sequences (resting state and four task-based scans) twice, once before (T1) and once at the end of the 18 h fasting period (T2, Table 3). The MRI acquisition is performed on a 3-Tesla whole body scanner (GE 750) with a 32-channel head coil. We also assess heart rate and breathing rate continuously during scanning to better control for possible artifacts during preprocessing of functional data. For more detailed description of acquisition parameters and the single tasks please refer to Supplementary Material (1.4, Additional file 1).

**Table 3:** Overview of sequences and acquired measures during the MRI session for timepoint 1 (T1) and timepoint 2 (T2). Established tasks were used for the investigation of reward processing [28], food processing [20], emotional face processing [95], and visual processing [66]

Scan	Measure	T1	T2
*Structural MRI*			
T1W	T1-weighted structural brain image	X	
DTI	Diffusion weighted brain image		X
*Functional MRI*			
Resting State	Resting state connectivity	X	X
Monetary reward task	Reward processing	X	X
Food viewing	Food cue reactivity	X	X
Fearful faces task	Emotional processing	X	
Houses task	General visual processing	X	

#### Endocrinology and metabolism

##### Blood

For assessment of the endocrine and metabolic response to fasting, blood samples are taken once prior to the 18-h fast (Sample 1) and twice during the 18-h fast on the second day (Sample 2, Sample 3 Table 4).

**Table 4:** Overview of endocrinological parameters measured from blood samples during three different timepoints

Analysis	Sample 1 Day 1 (T1) 3:30 pm	Sample 2 Day 2 8:00 am	Sample 3 Day 2 10:00 am	Sample type
***Hypothalamic–pituitary–adrenal axis***				
Adrenocorticotropic hormone (ACTH)	X	X	X	Plasma
Cortisol	X	X	X	Serum
***Hypothalamic–pituitary–thyroid axis***				
Thyroid-stimulating hormone (TSH)	(X)	X		Serum
Free triiodothyronine (fT3)	(X)	X		Serum
Free thyroxine (fT4)	(X)	X		Serum
***Hypothalamic–pituitary–gonadal axis***				
Follicle stimulating hormone (FSH)		X		Serum
Luteinizing hormone (LH)		X		Serum
Dehydroepiandrosterone (DHEAS)		X		Serum
Sex hormone-binding globulin (SHBG)		X		Serum
Testosterone		X		Serum
***Hypothalamic–pituitary–somatotropic axis***				
Insulin-like growth factor 1 (IGF-1)	X	X		Serum
Insulin-like growth factor-binding protein 1 (IGFBP-1)	X	X		Serum
Insulin-like growth factor-binding protein 3 (IGFBP-3)	X	X		Serum
***Gastrointestinal hormones***				
Acyl/deacyl-ghrelin	X	X	X	Plasma
***Adipokines***				
Adiponectin (ADIP)	X	X	X	Serum
Leptin		X		Serum
***Electrolytes***				
Sodium	X		X	Plasma
Sodium	X		X	Urine

##### Microdialysis

The direct metabolic response in subcutaneous adipose tissue to fasting is addressed in situ by microdialysis. A probe with a tubular semipermeable membrane is placed subcutaneously in the abdominal fat after Neuroimaging T1 (hour 9 day 1) [5, 49]. A sterile Ringer’s solution is continuously pumped through the dialysis tube at a flow rate of 0.5 μl/min. The dialysate samples are collected in microvials that are changed every 30 min. Overnight, one cumulative sample is collected between 23:00 and 6:00 to avoid disturbing the participant during sleep. The probe remains in place until after the last blood sampling on the second day. From the dialysate metabolites, including glucose, lactate pyruvate, and glycerol are analyzed for each participant repeatedly (CMA 600 Microdialysis Analyzer, CMA Microdialysis AB, Stockholm, Sweden). The data will provide us with information about glycolysis, gluconeogenesis, lipolysis with a high temporal resolution and their contribution to generate energy during the fasting period [5].

#### Intestinal microbiota

The stool samples are collected after the study visit at home, using OMNIgeneGut (OM-200, DNA Genotek) self-collection kits. Dietary intake for the 24 h before the stool sample collection is assessed via the Online 24-Hour Recall Log Book (Table 1). The samples are mailed to the laboratory to extract microbial genomic DNA. In order to obtain high-resolution taxonomic and functional microbiome data, we will perform whole genome shotgun sequencing. Raw sequence data will be quality filtered and trimmed to remove bases with Phred quality scores less than 20. Downstream bioinformatics analysis will consist of: i) taxonomic composition; ii) functional composition; iii) alpha diversity (as measured by Shannon index) and beta diversity (quantified by Bray Curtis and UniFrac metrics); and iv) computing descriptive statistics and identifying groups within the data, as well as performing statistical analyses between subgroups using additional metadata, where available [86]. Since the sequencing technology and bioinformatics tools are rapidly advancing, we will utilize the most suitable methods and tools available at the time of analysis. For more detail see Supplementary Material 1.6, Additional file 1.

#### Immunology

We measure concentrations of immune-related cytokines, cell injury, and cell death markers at three different timepoints (Table 4) in plasma or serum depending on the assay (Table 5). The cytokines are representative of the function of various immune cell populations and have been previously used in studies of psychiatric disorders, including depression, bipolar disorder, and schizophrenia [41]. We also gather information on cell injury and death by measuring concentrations of nuclear molecules and general cell loss markers, such as lactate dehydrogenase (LDH).

**Table 5:** Overview of immunological markers estimated from blood during day 1 and day 2

Analysis	Marker
***Cytokine markers***	Interleukin-1 receptor antagonist (IL-1RA)
	Interleukin 6 (IL-6)
	Interleukin 8 (IL-8)
	Tumor necrosis factor-α (TNF-α)
	Chemokine (C-C motif) ligand 5 (CCL5)/regulated on activation, normal T cell expressed and secreted (RANTES)
	Monocyte chemoattractant protein 1 (MCP1)
	High mobility group box 1 (HMGB1)
	Interleukin-1 receptor antagonist (IL-1RA)
***Cell death markers***	Double stranded deoxyribonucleic acid (dsDNA)
	Nucleosomes
***Cell injury markers***	
**General cell injury**	Lactate dehydrogenase (LDH)
**Liver cell injury**	Aspartate transaminase (AST)/alanine transaminase (ALT)
	Glutamate dehydrogenase (GLDH)
	α-fetoprotein (AFP)
	microRNA-122 (mRNA-122)
**Muscle cell injury**	Creatine kinase (CK)
**Single layer epithelial cell injury**	Total & cleared cytokeratin 18 (CK-18)

### Data management

#### General data security

All network communication between IT systems, including between participant or study operator devices, used in the study is encrypted by cryptographically strong methods as standard, if not otherwise specified. The hosting department provides central university network security services such as a central firewall and perimeter protection for the used networks. For more detail on data management please refer to Supplementary Material 1.6, Additional file 1.

#### Data storage and business logic

To handle administrative and research data during the collection phase of the study, we have implemented Karolinska Institutet Biobank IT, which is organizationally part of the core facility Karolinska Institutet Biobank. Data in file form are placed on the study research data disk area and indexed in a Central Research Database by an automated Study Data Management Application. The Central Research Database uses MS SQL Server technology, is only accessible by pre-specified users from selected department networks and has its own authentication scheme in addition to the department centralized directory service in the form of MS Active Directory.

All datasets will be stored in Sweden within in the described systems. All project investigators will be given access to their respective data sets and will have access to other sites data by request. To ensure confidentiality, data dispersed to project team members will be blinded of any identifying participant information.

#### Web survey platform

Confirmit is an online web survey platform service for study operators to implement and publish online web surveys and to perform data management for survey data collection. Confirmit allows us to implement surveys with advanced logical action flow, question filters, and scripted survey events. Study specific resources and collected survey data are only accessible by selected study operators. Additional PGP encryption, using a user-specific encryption key, is applied.

#### Web site

On the study website participants are authenticated with strong authentication methods for access to personal areas where they consent electronically to the study and access the web surveys. We use national electronic identity technology: Swedish BankID, or technology of similar capacity to perform authentication. The visual layout of the web site is made to illustrate progress through the study and the online web surveys to the participant.

#### Online 24-h recall web tool

The 24-h dietary recall logbook is introduced in the study through an online service by Nutrition Data Sweden AB. The 24-h recall logbook is a tool where the study participant can log nutrition intake before stool sample collection. The tool provides basic data quality protection of data entries made by the participants. Since being an online web tool, it is accessible through the internet. Study specific resources and collected survey data are only accessible by selected study operators.

### Statistical analyses

The MZ co-twin-control design is a method to evaluate the effect of non-shared factors on risk for AN, in the present study. Because MZ twins within a pair share, for all intents and purposes, 100% of their segregating genes, comparing twins discordant for AN controls for genetic and shared environmental factors. Thus, associations found between AN and other variables of interest suggest either a causal relationship, e.g., the variable of interest is a risk factor for AN or is a result of having AN, or a third variable indicative of an individual-specific factor that influences both AN and the variable of interest.

We will first contrast twins from discordant pairs (affected vs unaffected) on the wide array of variables evaluated in this study, which assesses state factors. Given that some of the outcomes for the present study are ordinal and some are repeated measures, and we anticipate using appropriate covariates in our models, we will apply mixed models. These models account for the clustered nature of twin data (i.e., non-independence of observations) and can incorporate fixed and random effects. In addition, the distribution of the outcome variables can be specified, and covariates can be added.

Second, the concordant unaffected MZ control twins will be included in the above models to evaluate whether having an affected co-twin influences the outcome variables. Results from these analyses may provide evidence that the co-twin’s affection status (an indication of familial influences of either environmental or possibly also epigenetic nature) is associated with the outcome if, e.g., the concordant unaffected twins have values or risk lower than those for the unaffected discordant twins, and these, in turn are lower than those for the affected discordant twins. Thus, these expanded models will be able to evaluate whether factors are state or trait in nature.

Those variables in which the discordant unaffected co-twins are significantly different than the affected twins will be further evaluated. In this case, the affected twins will be subdivided into those who are currently ill (state factors) and those who are recovered (“scar” effects). Pairwise comparisons will be made across the three groups: unaffected, recovered, and currently ill. “Scar” effects will be considered to be present if all three groups differ from one another (see Fig. 1; Table 6). While pairwise comparisons seem the right approach if subgroups constitute of equal (high) number of study participants, one also has to take into consideration that it might not be possible to clearly separate the group of affected twins into ill and recovered. In this case we propose a working model of using recovery status as continuous variable that can be controlled for as covariate in subsequent analyses. General covariates (e.g., illness duration, or nationality) appropriate to each domain will be entered into models. Analyses that will be across different domains (e.g., neuroimaging and endocrinology) will be handled with caution and covariates for both domains accounted for. Corrections for multiple testing will be considered within each domain to determine significance (e.g., for fMRI we will use the false discovery rate (FDR) or non-parametric permutation based approaches). Effect sizes and clinical relevance will be discussed based on the previous literature and current clinical practice.

**Table 6:** Overview over expected differences/similarities between subgroups. ≠ describes expected differences, = describes expected similarities between participants. Case Affected I = Case Twin affected from the disorder and currently ill. Case Affected R = Case Twin affected by the disorder and currently recovered, Case Unaffected = Monozygotic twin sister of the currently ill or recovered affected twin, Control = Healthy control twin

Parameter	Case Affected I vs Case Affected R	Case Affected I vs Case Unaffected	Case Affected R vs Case Unaffected	Case Affected I vs Control	Case Affected R vs Control	Case Unaffected vs Control
**State Parameter**	≠	≠	=	≠	=	=
**Trait Parameter**	=	=	=	≠	≠	≠
**Scar Parameter**	=	≠	≠	≠	≠	=

## Discussion

CREAT is one of the first studies of AN that will allow us to parse unique environmental, genetic, and medical contributions (e.g., sequelae of undernutrition) of the disorder and therefore advances the debate of identifying underlying risk factors across different domains [88]. The comprehensive investigation optimizes the natural experiment of MZ twins who are discordant for AN. This design allows us to address several key questions related to AN risk and recovery across a broad range of relevant domains. In the following section we discuss both advantages and limitations of the current research protocol.

Adequate sample sizes are only attainable in locations with extensive twin registries, such as Sweden or Denmark. The study is demanding for participants and we have implemented a number of measures to ensure comfort and to express our deep appreciation of all participants. In addition to monetary incentives, twins are accompanied throughout the study by a dedicated research nurse and at night by specially trained nursing assistants. We have built in digital incentives and progress markers as participants work their way through questionnaires, and we are working to create a CREAT community through study branding, staying in contact with participants, and sharing results. In so doing, we hope to be able to return to twins in the future should additional research questions arise.

The specific research methods in each of the areas of the study were chosen by a panel of experts to maximize the utility of this specific data set. For example in the area of brain imaging well-validated paradigms [20, 28, 31, 66, 95] as well as state of the art statistical methods were chosen in order to ensure comparability with existing neuroimaging studies and excellent data quality. As a result of overly simplistic descriptions of the brain connectome in previous resting state research [38], we intend to apply multivariate techniques [17], graph theoretical approaches [109], and dynamic effective connectivity analyses [95], all of which offer promising tools to ask questions that go beyond simple connectivity analyses with resting state data [11, 39, 40] and integrate results from the other study domains, e.g. metabolic and endocrinological data.

Previous neurocognitive studies have often investigated the subcomponents of (value-based) decision making within single tasks (e.g., Wisconsin card sorting task, Iowa gambling task) that are unable to further differentiate between more specific concepts such as risk aversion and loss avoidance. Hence, the task battery employed for this project [90] also provides measures for risk aversion (for gains), risk seeking (for losses), as well as loss aversion. In a similar vein, other neurocognitive abilities as well as body image distortion (i.e., Somatomap [94]) are assessed using validated state-of-the-art task batteries. We are aware of the problem of learning effects when applying these tasks repeatedly. Since the neurocognitive domain includes established and validated tasks, there is no easy solution to this problem, without possibly changing the difficulty or complexity of the task, or extending the protocol beyond what is feasible in one session. However, since the learning effects will also be visible in the HC twins, we will focus only on the relative differences (e.g., between the AN discordant twins accounting for differences that were also observable in HC twins).

Methods in the field of genetics, epigenetics, microbiome research, and immunology develop rapidly. Analytic strategies will be finalized considering the latest technologies and methodologies available at completion of data collection. However, we have taken great care to assess all potential confounds and to store and preprocess the biological materials under conditions that ensure the integrity of the materials. New publicly available databases and computational methods will help us to enrich our findings, control for population-specific effects, and identify relevant metabolic and immunological pathways [50]. Furthermore, given the deep phenotyping of CREAT, we will be able to test for associations and interactions between genetic and immunological markers, hormones, and alterations of the microbiome and potentially answer the current questions regarding the gut-brain axis [105].

CREAT does pose logistical challenges and results will have limitations. Firstly, our strict inclusion criteria (i.e., requiring rigorous discordance) will probably lengthen the recruitment process to ascertain eligible pairs. Secondly, although the study itself will be conducted at a single site, participant recruitment covers both Sweden and Denmark, possibly introducing subtle differences in recruitment procedures, interviewing, and translations and wordings that might affect the comparability of the data (which we will control for statistically). In long-running studies, above all at different recruitment sites, it is essential to ensure that all staff are highly trained, and procedures remain consistent across the study period (i.e., do not evidence drift). Thirdly, the targeted sample size is relatively small. Although we have power to detect relatively small effect sizes, other clinical variables may have an adverse effect on power. For example, it is not possible to predict in advance the exact proportion of participants who are actively ill versus recovered. Depending on the distribution, we may be underpowered to detect differences between these two subgroups. Furthermore, duration of illness might moderate some of the expected differences (and will therefore be treated as a covariate in statistical models). Also, including only females within a specified age range, might yield a limited picture of the disorder. This decision is practical as we were unable to identify discordant MZ male twins in the register and it is essential that we only study twins who have passed through the age of risk to ensure unaffected status. Lastly, similar to other complex studies [76], planned analyses represent a mixture of hypothesis-driven and exploratory research questions. The exploratory aims emerge from findings in the literature regarding where differences may lie in affected versus unaffected individuals.

With these challenges and limitations in mind, we have instituted multiple checks including extensive standard operating procedures (SOPs) and detailed protocols to ensure the longevity of the project and maximize the utility of the rich data that will be captured from these highly valuable sets of twins.

The twin design employed in the current study offers the opportunity to deliver a comprehensive understanding of possible alterations within genetics, epigenetics, neurocognitive function, neuroimaging, metabolism, endocrinology, microbiology, and immunology in AN by disaggregating genetic and environmental factors influencing risk for the disorder.

## Supplementary information

**Additional file 1.** Supplementary information about diagnostics and measured parameters in the study.

## Data Availability

Not applicable.

## Abbreviations

ACTH: Adrenocorticotropic hormone
ADIP: Adiponectin
AFP: α-fetoprotein
ALT: Alanine transaminase
AN: Anorexia nervosa
AQ-10: Autism spectrum disorder
AST: Aspartate transaminase
BFI: Big five inventory
BIS/BAS: Behavioral inhibition and behavioural activation system
BMI: Body-mass index
BOLD: Blood oxygenation level-dependent
CCL5: Chemokine (C-C motif) ligand
CK: Creatine kinase
CREAT: Comprehensive risk evaluation for anorexia nervosa in twins
CRP: C-reactive protein
DHEAS: Dehydroepiandrosterone
dsDNA: Double stranded deoxyribonucleic acid
DSM-V: Diagnostic and Statistical Manual of Mental Disorders
DTI: Diffusion tensor imaging
DTR: Danish twin registry
DXA: Dual X-ray absorptiometry
ED: Eating disorder
ED100K: Eating disorder diagnostic online questionnaire
EDE: Eating Disorder Examination
EDE-Q: Eating Disorder Examination Questionnaire
fMRI: Functional magnetic resonance imaging
FSH: Follicle stimulating hormone
FT3: Free triiodothyronine
FT4: Free thyroxine
GAD-7: General anxiety disorder questionnaire
GLDH: Glutamate dehydrogenase
GM: Grey matter
GWAS: Genome-wide association study
HMGB: High mobility group
HMRH: Hormonal, menstrual and reproductive history
IGF: Insulin-like growth factor
IGFBP: Insulin-like growth factor-binding protein
IL: Interleukin
IL-1RA: Interleukin-1 receptor antagonist
IQ: Intelligence quotient
LDH: Lactate dehydrogenase
LH: Luteinizing hormone
MCP: Monocyte chemoattractant protein
MINI: Mini International Neuropsychiatric Interview
MPS: Multidimensional perfectionism scale
MR(I): Magnetic resonance (imaging)
MZ: Monozygotic
OCI-R: Obsessive compulsive inventory – revised
PANAS: Positive and negative affect scale
PHQ9: Patient health questionnaire
POTS: Perception of teasing scale
QOLI: Quality of life inventory
Ravens SPM: Raven’s progressive matrices und vocabulary scales
ROI: Region of interest
SBLUP: summary data-based best linear unbiased predictors
SCID: Structured Clinical Interview for DSM-5
SD: Standard Deviation
SHGB: Sex hormone-binding globulin
SPM: Statistical parametric mapping software
STR: Swedish twin registry
SUPPS-P: Impulsive behaviour scale
T1: Timepoint 1
T2: Timepoint 2
TNFα: Tumor necrosis factor α
TSH: Thyroid-stimulating hormone
VAS: Visual analogue scale
VBDM: Value based decision making
WM: White matter
