# Supplementary material for: Study protocol of comprehensive risk evaluation for anorexia nervosa in twins (CREAT): a study of discordant monozygotic twins with anorexia nervosa
Source: BMC Psychiatry. 2020 Oct 14;20:507. doi: 10.1186/s12888-020-02903-7 (PMC7557028; doi:10.1186/s12888-020-02903-7)
Supplement: Supplementary file 1 — Additional file 1. Supplementary information about diagnostics and measured parameters in the study. [file 12888_2020_2903_MOESM1_ESM.docx]

**Supplementary Material**

1. **Method**
   1. Screening Procedure

For verification of zygosity, saliva samples are collected using Isohelix kits. The use of saliva kits is a standard proven technology used on hundreds of thousands of samples worldwide and yielding ample DNA for zygosity testing and for follow-up analyses. The twins are sent an Oragene™ saliva kit. The samples are typed for 44 autosomal and three X-chromosome SNPs. We require a <95% SNP genotyping performance per individual and fully matching SNP and phenotypic sex. A maximum of 2 missing genotypes per pair are allowed. We allow for one potential genotyping error or a mismatch between twins per batch of 20 samples.

- 1. Questionnaires
     1. *MiniMeal-Q*

Eating behaviors are assessed with the MiniMeal-Q (Christensen et al., 2013), a validated interactive web-based assessment that includes questions regarding 75-126 food items, depending on the number of follow-up questions. MiniMeal-Q assesses intake of food items, dishes, and beverages, which enables the calculation of energy and nutrient intake (including alcohol). It also asks about meal patterns; eating behavior, such as restaurant visits; intake of fast food, light products, probiotics, the use of cooking fat and salt, as well as the use of dietary supplements.

- - 1. *Impulsive behavior scale (SUPPS-P)*

The SUPPS-P (Whiteside et al., 2005; Whiteside & Lynam, 2001) is a short 20 item version of the original 59-item self-report that assesses five subscales (urgency, premeditation, perseverance, sensation seeking, and positive urgency) that are used to measure five distinct dimensions of impulse behavior in adolescents and adults (ages 12 and older). It is designed to measure impulsivity across dimensions of the Four Factor Model of personality: Premeditation (lack of), Urgency, Sensation Seeking, Perseverance (lack of). Respondents are asked to rate their behavior and attitudes on a 4-point scale from agree strongly to disagree strongly.

- - 1. *Active-Q*

The Active-Q is a web-based questionnaire that assesses total physical activity and inactivity in adults (Bonn et al., 2012). The Active-Q assesses habitual activity during the past year via questions in four different domains: (1) daily occupation, (2) transportation to and from daily occupation, (3) leisure time activities, and (4) sporting activities.

- - 1. *Eating Disorders Examination—Questionnaire Version 6 (EDE-Q)*

The EDE-Q-6 (Fairburn, 2008) consists of 28 items; 10 minutes and is based on and correlates well with the EDE. There are general and clinical population norms for EDE-Q, for young adult women in Sweden (Welch et al., 2011).

- - 1. *Quality of life Inventory (QOLI)*

The QOLI; (Frisch et al., 1992) measures quality of life within 16 areas; health, self-respect, goals and values, economy, work, leisure, learning, creativity, helping others, love, friends, children, relatives, home, community, and society. The respondent is first asked to rate on a scale from 0-2 how important each area is, and then on a scale from -3 to 3 (where 0 is not an alternative) how satisfied they are with that area.

- - 1. *Fagerström Test for nicotine dependency scale*

The Fagerström Test for Nicotine Dependence (Heatherton et al., 1991) is a standard instrument for assessing the intensity of physical addiction to nicotine. The test was designed to provide an ordinal measure of nicotine dependence related to cigarette smoking. It contains six items that evaluate the quantity of cigarette consumption, the compulsion to use, and dependence.

- - 1. *Hormonal, menstrual and reproductive history (HMRH)*

The questionnaire assesses hormonal, menstrual and reproductive history and is based on an extensive online questionnaire (Gabrielson et al., 2017).

- - 1. *Perception of Teasing Scale (POTS)*

The POTS (Thompson et al., 1995) consists of 11 items and measures general weight- and competency teasing. For each item, the respondent is asked to rate how often they thought they were teased (never to always) and how upset they were by the teasing (not upset to very upset).

- - 1. *Edinburgh Handedness Inventory*

The Edinburgh Handedness Inventory (Oldfield, 1971) assesses via self-report the dominance of a person's right or left hand in everyday activities, sometime referred to as laterality which can be an important factor regarding brain structure and function.

- - 1. *Behavioral inhibition/approach system (BIS/BAS)*

The BIS/BAS (Carver & White, 1994) measures two dimensions with 24 items. The behavioral approach system (BAS) is believed to regulate appetitive motives, in which the goal is to move toward something desired. The behavioral avoidance (or inhibition) system (BIS) is said to regulate aversive motives, in which the goal is to move away from something unpleasant.

- - 1. *Frost Multidimensional Perfectionism Scale (MPS)*

The MPS (Frost et al., 1990) consists of 12 items and six subscales (concern over mistakes, doubts about actions, personal standards, organization, parental criticism, and parental expectations). The 12 items were previously selected by Bulik and colleagues (Bulik et al., 2003) out of the full MPS based on research findings and communication with the scale developers.

- - 1. *Patient Health Questionnaire (PHQ-9)*

The PHQ-9 (Spitzer et al., 1999) is a multipurpose instrument for screening, diagnosing, monitoring, and measuring the severity of depression.

- - 1. *Generalized Anxiety Disorder 7-item scale (GAD-7)*

The GAD-7 (Spitzer et al., 2006) is a 7-item instrument used to briefly measure or assess one of the most common mental disorders. The cut off points for mild is a score of 5, for moderate is 10 and for severe anxiety is a score of 15. When using this screening tool, further evaluation is recommended when there is a score of 10 or higher. The GAD-7 is moderately good at screening three other common anxiety disorders like panic disorders, social anxiety disorder, and post-traumatic stress disorder.

- - 1. *Autism Spectrum Quotient 10 (AQ-10)*

The AQ-10 (Baron-Cohen et al., 2001) tool is recommended for use with adults with possible autism who do not have a moderate or severe learning disability. This may help identify whether an individual should be referred for a comprehensive autism assessment.

- - 1. *Obsessive Compulsive Inventory - Revised (OCI-R)*

The OCI-R (Foa et al., 2002) is a self-report instrument consisting of 18 items comprising 6 sub-scales, which measures severity of obsessions and compulsions. Each item is rated on a 5-point Likert scale of symptom frequency and distress.

- - 1. *Big Five Inventory (BFI)*

The BFI (Goldberg, 1990) is a 44-item personality test measuring extraversion, openness, agreeableness, neuroticism, and conscientiousness.

- - 1. *24-hour Diet Recall*

The 24-hour recall logbook is an online tool where the study participant can log nutrition intake on the internet. Participants are asked to fill the 24-hour diet recall 24 hours before they submit their stool sample.

- - 1. *Visual analogue scale (VAS)*

Participants are asked on 11 occasions how hungry and thirsty they feel (four items), as well as how tired (one item) and anxious (one item). The answers are given on a visual analogue scale (slider) ranging from ‘very’ to ‘not at all’. The questionnaire is applied at baseline, before MRI day 1, after MRI day 1, before dinner, after dinner, after getting up, before MRI day 2, after MRI day 2, before brunch, after brunch.

- - 1. *Positive and negative affect scale (PANAS)*

The PANAS is a self-report questionnaire that consists of two 10-item scales to measure current positive and negative affect. The items are rated on a 5-point Likert scale ranging from ‘very’ to ‘not at all’. The PANAS is administered on six occasions after the VAS, at baseline, after dinner, before going to bed, after waking up, and after brunch.

- - 1. *Antibiotics & gastrointestinal symptoms*

The self-designed questionnaire assessed whether participants have experienced or not one or several of the following gastrointestinal symptoms during the last three months: constipation, diarrhea, flatulence, stomach ache or abdominal pain, feeling uncomfortable after meals, pyrosis, pain or burning feeling in the chest. Further, participants are asked to indicate the texture and shape of their stool, via multiple choice. Additionally intake of medication including antibiotics during the last three months is assessed.

- 1. Cognitive Functions
     1. *Set-shifting*

To assess individual differences in set-shifting, we follow the approach taken by other authors in previous work on individual differences in executive functioning (Friedman et al., 2008; Miyake et al., 2000; Wolff et al., 2016). All participants complete a battery of three computerized set-shifting tasks during two single sessions with an approximate total duration of 30 min.

All three set-shifting tasks (number-letter, color-shape, category switching) require participants to give speeded responses while maintaining accuracy. Performance is quantified by outcome measures based on error rate (ER) and reaction time (RT) which are used to calculate inverse efficiency scores (IESs; (Bruyer & Brysbaert, 2011; Townsend & Ashby, 1983). As all tasks comprise two subtasks, thus, there are “switch” trials (where the task set is different from the preceding trial) and “no-switch” trials (where the task set is the same as in the preceding trial). Switch cost are calculated as the IES difference between “switch” and “no-switch” trials.

- - 1. *Value-based decision making (VBDM)*

Several different tasks have been used to measure value-based decision making in AN, a construct that by itself comprises several sub-processes. However, most standard tasks do not distinguish these sub-processes. Therefore, it has become increasingly desirable to measure choice behavior in a variety of contexts with improved, precise, and consistent methods. The proposed task battery for this project (Pooseh et al., 2018) investigates four subcomponents: delay discounting, loss aversion during probability discounting to gains and losses as well as risk aversion during a mixed gambling task using an adaptive Bayesian algorithm. These qualities are encoded in a parameter called kdd, kpdw, kpdl, for the tasks called delay discounting, probabilistic discounting for win and probabilistic discounting for loss respectively and λ for the mixed gamble task. The consistency of each participant, or coherence with their previous choices, is encoded in parameters called βdd, βpdw, βpdl, βmga.

- - 1. *Rey Osterrieth Complex Figures Test (RCFT)*

The RCFT is a widely used neuropsychological assessment tool in which the respondents are first asked to reproduce a complicated line drawing as accurately as they can, first by copying the figure and then drawing it from memory (direct and delayed recall). The drawing style used by the respondent is used to measure central coherence abilities. An index, called the central coherence index (CCI) is calculated. Higher CCI score is indicative of a more global processing strategy.

- - 1. *Somatomap 3D*

Somatomap is a body image distortion measurement tool (Ralph-Nearman et al., 2019). It uses gender-specific three-dimensional (3D) body avatars, in which different body areas may be sculpted from all angles to replicate one’s current perceived, as well as ideal, body shape. Comparisons are made between subjective vs. actual body measurements (5 areas: bust, right bicep, waist, hips, and right thigh), to provide regional and total quantifications of body image distortions.

- - 1. *Raven’s Standard Progressive Matrices (SPM)*

Raven's SPM is a nonverbal test for measuring abstract reasoning. It consists of 60 items, each with a visual geometric design with a missing piece. For each item/task, subjects are asked to choose the most suitable piece among 6-8 alternatives. Raven's SPM is generally viewed a non-verbal measure of fluid intelligence, as it more specifically measures cognitive ability in terms of cognitive reasoning.

- 1. Neuroimaging
     1. *Brain structure*

T1-weighted structural brain scans are measured at T1. The T1-weighted structural brain scans for morphometric analyses and as a reference for functional and diffusion data are acquired with rapid acquisition gradient echo (MP-RAGE) sequence with 1mm isotropic resolution and an acquisition time of 6:01min. Diffusion weighted images (DTI) are measured at T2. DTI is obtained using a multi-band spin-echo sequence with 60 diffusion directions, b=1500s/mm2 with 2.3mm isotropic resolution acquired in 5:31min. Additionally an image with inverted phase-encoding is acquired in 47s to correct for distortions.

The structural T1 images are processed with the automated volumetric segmentation and cortical surface reconstruction software FreeSurfer to measure vertexwise cortical thickness (CT) and volumes of anatomical labels [http://surfer.nmr.mgh.harvard.edu, see (King et al., 2015) for details]. Furthermore we estimate shape characteristics and graph theory metrics from cortical thickness across the brain to characterize structural brain network properties.

For the DTI images a tensor fitting method is used and the level of fractional anisotropy (FA) and further white matter (WM) integrity metrics (AD, RD, MD) are computed (Chang et al., 2005) . The WM tracts of the brain networks are reconstructed by using the deterministic fiber tracking, based on the FACT (fiber assignment by continuous tracking) algorithm (Mori & van Zijl, 2002). The number of streamlines between two nodes (e.g., defined using FreeSurfer regions of interest) serve as the connectivity strength for graph theoretical analysis. Using these matrices, weighted, undirected graph networks are constructed. Global and local network metrics, including characteristic path length, degree and clustering coefficient are derived using functions from the BCT toolbox (http://www.nitrc.org/projects/bct/).

- - 1. *Brain function*

fMRI data can be obtained during specific tasks or during rest. Resting state functional connectivity (RSFC) is measured at both timepoints T1 and T2. The functional images during rest (eyes open, looking at a black screen) are acquired for 8 min by using a gradient-echo T2*-weighted multi-band echo planar imaging with 2mm isotropic resolution and a TR of 2s for higher temporal resolution. The field of view is tilted 15° towards AC–PC line (to reduce signal dropout in orbitofrontal regions).

There are four task-based fMRI measurements. 1) Food cue reactivity, during which the participant views 100 pictures of food (50 high caloric and 50 low caloric) and 50 non-food pictures as described previously (Sanders et al., 2015). Pictures are presented in a block design with high caloric and low caloric food- and non-food blocks in six different pseudorandom orders to ensure that none of the conditions is presented twice in a row. 2) Reward anticipation, during which participants perform an instrumental motivation task, an adjusted version of the common monetary incentive delay paradigm (Knutson et al., 2001), while lying in the MRI scanner. Both tasks are presented during T1 and T2. 3) General visual processing using house images (Li et al., 2015). In order to specifically test detailed and configural/holistic visual processing, we use digital photographs that are spatial-frequency filtered to contain only high spatial frequency (HSF) and low spatial frequency (LSF) visual elements, respectively (Feusner et al., 2011). We also use unaltered, normal spatial frequency (NSF) photographs. Squares/rectangles serve as control images. During the presentation, participants perform a forced-choice, 2-alternative matching task of pressing a button corresponding to which of two images match the target image in the top half of the screen. 4) Face processing during which we use validated and robust stimuli consisting of faces with fearful expressions to test limbic reactivity, habituation, and connectivity (Vuilleumier & Pourtois, 2007). The first half tests habituation/sensitization (Ishai et al., 2004). The second half tests internal modulation of limbic activity associated with emotion labeling (Beesdo et al., 2009; Rangaprakash et al., 2018). Tasks 3 and 4 are only measured at T1.

For all four task-based fMRI scans we use a standard EPI with 2mm isotropic resolution and a TR of 2 seconds using a 32-channel head coil. The field of view is tilted 15° towards AC–PC line (to reduce signal dropout in orbitofrontal regions).

Functional images of the fMRI task paradigms and RSFC are processed using SPM12 toolbox (http://www.fil.ion.ucl.ac.uk/spm/) within the Nipype framework (http://nipy.org/nipype/), with the exception of the visual processing and fearful face processing tasks, which are processed using FEAT (FMRI Expert Analysis Tool) version 6.0, part of FSL (FMRIB’s Software Library; http://www.fmrib.ox.ac.uk/fsl). The quality of the fMRI data is ensured by manual inspection, using artifact detection tools [ART (Whitfield-Gabrieli et al., 2009)] and a stringent motion control. Artifacts related to heart pulsatility and breathing are corrected using the RETROICOR package (Glover et al., 2000). The slice time corrected functional data are realigned, registered and normalized to MNI using a group-specific template. For RSFC data DPARSFA toolbox (Chao-Gan & Yu-Feng, 2010) is applied for temporal filtering and regression of nuisance covariates (WM, cerebrospinal fluid(CSF)). The resulting volumes are analyzed using high-dimensional independent component analysis (ICA, see Boehm et al. (2014) for details), joint ICA (Sui, Adali, Yu, Chen, & Calhoun, 2012) to also include GM and WM measures and, in a parallel processing stream, parcellated into ROIs (e.g. empirically via ICA, with standard approaches) for graphical analysis. Network metrics, including characteristic path length, degree and clustering coefficient will be derived using functions from the BCT toolbox (Rubinov & Sporns, 2010).

- 1. Intestinal microbiota

Stool sampling allows for characterization of changes in the intestinal microbiota related to food intake and nutritional status. A stool collection kit (OMNIgene.Gut) containing a preservative that stabilizes DNA at ambient temperature is given to the participants at the end of the second study day at the Karolinska Institutet. Participants are then asked to contribute a sample approximately two weeks after the study. The home collected samples are kept at room temperature until mailed to Karolinska Institutet biobank where the samples are stored at -80°C until used. After removal from storage, stool microbial genomic DNA will be isolated and amplified using the most suitable methods available at time of the analysis. Whole genome sequencing will be conducted to achieve species-level resolution and functional profiling in affected versus discordant unaffected co-twins (state-related alterations), unaffected discordant twins and twin controls (trait-related changes), and affected recovered twins versus unaffected discordant co-twins (scar-related alterations). Metagenomic DNA sequence reads will be decontaminated from human genome reads using KneadData (<http://huttenhower.sph.harvard.edu/kneaddata>). The resultant sequencing data will be analyzed by read-based profiling using tools such as Kraken (Wood & Salzberg, 2014) and HUMAnN (Franzosa et al., 2018). Higher-level analyses including alpha and beta diversity using the vegan package in the R statistical program, and taxonomic profiling will be subsequently performed to find overall patterns in microbiome variation. Pearson's correlations will be used to test associations between variables with normal distributions; otherwise, Spearman's correlation will be applied. We will adjust for important covariates including but not limited to age and diet. Since the sequencing technology and bioinformatics tools are rapidly advancing, we will utilize the most suitable methods and tools available at the time of analysis.

- 1. Data management
     1. *Karolinska Institutet Biobank IT*

The Platform primarily consists of these main IT system components and technologies: 1. CRM system, 2. LIMS, 3. CMS, 4. Technical platform business routines, 5. Relational databases.

The CRM system Espo CRM is a tool for study operators to manage study administrative data, such as study participant data. The LIMS Labware LIMS is a formalized system for study operators to manage lab and sample data. The web CMS Wordpress is a tool for study operators to manage structured web sites and their content, such as pages, articles, and text entries. Data storage is provided by a set of databases using mainly MS SQL Server technology. These databases serve the CRM system, LIMS, web CMS, and the technical platform business routines. The Karolinska Institutet Biobank IT Platform also uses the Swedish BankID identification service to authenticate study participants and relate them to their Swedish National Identification Number (personnummer). The BankID service is a highly secure authentication and identification technology that is well established in Sweden among administrative authorities, other public sector organizations such as healthcare providers, and private sector companies such as banks and financial service providers.

- - 1. *Confirmit*

The set of questionnaires contained in the study survey battery are implemented in the online web survey platform Confirmit. Each survey instance related to a specific participant is secured by a unique GUID-like identifier string presented to the Confirmit platform by the Karolinska Institutet Biobank IT Platform website.

Collected survey data are automatically extracted through either the Confirmit FTP service, or through the Confirmit online API. Automated survey data extraction is implemented by the Automated Study Data Management Application. Survey data is stored in the Central Research Database in a format that reflects the original survey definition as much as possible.

- - 1. *Basic & Anthropometric measures*

Accelerometer measurements: Accelerometer data are manually extracted from the accelerometer recording devices by a study operator, using the corresponding accelerometer reader and the software GENEActiv. The stored files go through basic automated quality control by the Automated Study Data Management Application. Basic quality control of the data is also done through the GENEActiv software.

DXA measurements: DXA result data are personally handed over to the study personnel on a DVD-disc from the DXA-center. The disc contains a dfx-file importable by the GE Lunar Prodigy Encore software application. The dfx-file is manually copied into the study Central File Storage and imported into any study specific GE Lunar Prodigy Encore database if any is set up. The stored files go through basic automated quality control by the Automated Study Data Management Application. Further manual data inspection and quality control is done through the GE Lunar Prodigy Encore software application.

- - 1. *Neuropsychologial measures*

The set-shifting and VBDM batteries are executed on a study laptop. Saved logfiles with behavioral data are manually quality controlled and then stored in the study Central File Storage. They also go through a basic automated quality control by the Automated Study Data Management Application.

The Raven’s SPM is administered according to the standard instructions for individual testing, and the results are entered in the corresponding paper protocol. Data from the protocol are then entered into the Karolinska Institutet Biobank IT database.

- - 1. *Neuroimaging measures*

Task data: Behavioral task data of the imaging paradigms as well as physiological data are collected on a local computer at the imaging center. The logfiles are then manually quality controlled and then stored in the study Central File Storage. They also go through a basic automated quality control by the Automated Study Data Management Application.

Imaging data: Imaging files are in DICOM format and saved together with an identifying session ID number. The imaging files are first pushed manually to a temporary secure PACS server while the identifying session ID number is recorded in the Karolinska Institutet Biobank IT LIMS. The Automated Study Data Management Application uses the communication bus towards the biobank’s technical platform business routines to fetch newly entered session ID’s together with the corresponding study person ID to identify files to fetch from the PACS server and to relate those to the right study person. The imaging files are then automatically fetched by the Automated Study Data Management Application, go through a specific imaging quality control routine, and are finally stored in the study Central File Storage.

- - 1. *Metabolic and endocrine measures*

All endocrine samples are shipped to, processed, and stored at Karolinska Institutet biobank. Standard procedures and protocols for transport, handling, and storage of the samples have already been established in collaboration with the biobank.

**References**

Baron-Cohen, S., Wheelwright, S., Skinner, R., Martin, J., & Clubley, E. (2001). The autism-spectrum quotient (AQ): Evidence from asperger syndrome/high-functioning autism, malesand females, scientists and mathematicians. *Journal of Autism and Developmental Disorders*, *31*(1), 5–17.

Beesdo, K., Lau, J., Guyer, A., & et al. (2009). Common and distinct amygdala-function perturbations in depressed vs anxious adolescents. *Archives of General Psychiatry*, *66*(3), 275–285. https://doi.org/10.1001/archgenpsychiatry.2008.545

Bonn, S. E., Lagerros, Y. T., Christensen, S. E., Möller, E., Wright, A., Sjölander, A., & Bälter, K. (2012). Active-Q: Validation of the web-based physical activity questionnaire using doubly labeled water. *Journal of Medical Internet Research*, *14*(1).

Bruyer, R., & Brysbaert, M. (2011). Combining speed and accuracy in cognitive psychology: Is the inverse efficiency score (IES) a better dependent variable than the mean reaction time (RT) and the percentage of errors (PE)? *Psychologica Belgica*, *51*(1), 5–13.

Bulik, C. M., Tozzi, F., Anderson, C., Mazzeo, S. E., Aggen, S., & Sullivan, P. F. (2003). The relation between eating disorders and components of perfectionism. *American Journal of Psychiatry*, *160*(2), 366–368.

Carver, C. S., & White, T. L. (1994). Behavioral inhibition, behavioral activation, and affective responses to impending reward and punishment: The BIS/BAS Scales. *Journal of Personality and Social Psychology*, *67*(2), 319–333. https://doi.org/10.1037/0022-3514.67.2.319

Chang, L.-C., Jones, D. K., & Pierpaoli, C. (2005). RESTORE: Robust estimation of tensors by outlier rejection. *Magnetic Resonance in Medicine*, *53*(5), 1088–1095. https://doi.org/10.1002/mrm.20426

Chao-Gan, Y., & Yu-Feng, Z. (2010). DPARSF: A MATLAB Toolbox for “Pipeline” Data Analysis of Resting-State fMRI. *Frontiers in Systems Neuroscience*, *4*. https://doi.org/10.3389/fnsys.2010.00013

Christensen, S. E., Möller, E., Bonn, S. E., Ploner, A., Wright, A., Sjölander, A., Bälter, O., Lissner, L., & Bälter, K. (2013). Two new meal-and web-based interactive food frequency questionnaires: Validation of energy and macronutrient intake. *Journal of Medical Internet Research*, *15*(6).

Fairburn, C. G. (2008). *Cognitive behavior therapy and eating disorders*. Guilford Press.

Feusner, J. D., Hembacher, E., Moller, H., & Moody, T. D. (2011). Abnormalities of object visual processing in body dysmorphic disorder. *Psychological Medicine*, *41*(11), 2385–2397.

Foa, E. B., Huppert, J. D., Leiberg, S., Langner, R., Kichic, R., Hajcak, G., & Salkovskis, P. M. (2002). The Obsessive-Compulsive Inventory: Development and validation of a short version. *Psychological Assessment*, *14*(4), 485.

Franzosa, E. A., McIver, L. J., Rahnavard, G., Thompson, L. R., Schirmer, M., Weingart, G., Lipson, K. S., Knight, R., Caporaso, J. G., Segata, N., & Huttenhower, C. (2018). Species-level functional profiling of metagenomes and metatranscriptomes. *Nature Methods*, *15*(11), 962–968. https://doi.org/10.1038/s41592-018-0176-y

Friedman, N. P., Miyake, A., Young, S. E., DeFries, J. C., Corley, R. P., & Hewitt, J. K. (2008). Individual differences in executive functions are almost entirely genetic in origin. *Journal of Experimental Psychology: General*, *137*(2), 201.

Frisch, M. B., Cornell, J., Villanueva, M., & Retzlaff, P. J. (1992). Clinical validation of the Quality of Life Inventory. A measure of life satisfaction for use in treatment planning and outcome assessment. *Psychological Assessment*, *4*(1), 92.

Frost, R. O., Marten, P., Lahart, C., & Rosenblate, R. (1990). The dimensions of perfectionism. *Cognitive Therapy and Research*, *14*(5), 449–468.

Gabrielson, M., Eriksson, M., Hammarström, M., Borgquist, S., Leifland, K., Czene, K., & Hall, P. (2017). Cohort profile: The Karolinska mammography project for risk prediction of breast cancer (KARMA). *International Journal of Epidemiology*, *46*(6), 1740–1741g.

Glover, G. H., Li, T.-Q., & Ress, D. (2000). Image-based method for retrospective correction of physiological motion effects in fMRI: RETROICOR. *Magnetic Resonance in Medicine*, *44*(1), 162–167. https://doi.org/10.1002/1522-2594(200007)44:1<162::AID-MRM23>3.0.CO;2-E

Goldberg, L. R. (1990). An alternative" description of personality": The big-five factor structure. *Journal of Personality and Social Psychology*, *59*(6), 1216.

Heatherton, T. F., Kozlowski, L. T., Frecker, R. C., & Fagerstrom, K.-O. (1991). The Fagerström test for nicotine dependence: A revision of the Fagerstrom Tolerance Questionnaire. *British Journal of Addiction*, *86*(9), 1119–1127.

Ishai, A., Pessoa, L., Bikle, P. C., & Ungerleider, L. G. (2004). Repetition suppression of faces is modulated by emotion. *Proceedings of the National Academy of Sciences*, *101*(26), 9827–9832. https://doi.org/10.1073/pnas.0403559101

King, J. A., Geisler, D., Ritschel, F., Boehm, I., Seidel, M., Roschinski, B., Soltwedel, L., Zwipp, J., Pfuhl, G., Marxen, M., Roessner, V., & Ehrlich, S. (2015). Global cortical thinning in acute anorexia nervosa normalizes following long-term weight restoration. *Biological Psychiatry*, *77*(7), 624–632. https://doi.org/10.1016/j.biopsych.2014.09.005

Knutson, B., Adams, C. M., Fong, G. W., & Hommer, D. (2001). Anticipation of increasing monetary reward selectively recruits nucleus accumbens. *The Journal of Neuroscience: The Official Journal of the Society for Neuroscience*, *21*(16), RC159.

Li, W., Lai, T. M., Loo, S. K., Strober, M., Mohammad-Rezazadeh, I., Khalsa, S., & Feusner, J. (2015). Aberrant early visual neural activity and brain-behavior relationships in anorexia nervosa and body dysmorphic disorder. *Frontiers in Human Neuroscience*, *9*. https://doi.org/10.3389/fnhum.2015.00301

Miyake, A., Emerson, M. J., & Friedman, N. P. (2000). Assessment of executive functions in clinical settings: Problems and recommendations. *Seminars in Speech and Language*, *21*, 0169–0183.

Mori, S., & van Zijl, P. C. M. (2002). Fiber tracking: Principles and strategies - a technical review. *NMR in Biomedicine*, *15*(7–8), 468–480. https://doi.org/10.1002/nbm.781

Oldfield, R. C. (1971). The assessment and analysis of handedness: The Edinburgh inventory. *Neuropsychologia*, *9*(1), 97–113.

Pooseh, S., Bernhardt, N., Guevara, A., Huys, Q. J., & Smolka, M. N. (2018). Value-based decision-making battery: A Bayesian adaptive approach to assess impulsive and risky behavior. *Behavior Research Methods*, *50*(1), 236–249.

Ralph-Nearman, C., Arevian, A. C., Puhl, M., Kumar, R., Villaroman, D., Suthana, N., Feusner, J. D., & Khalsa, S. S. (2019). A Novel Mobile Tool (Somatomap) to Assess Body Image Perception Pilot Tested With Fashion Models and Nonmodels: Cross-Sectional Study. *JMIR Mental Health*, *6*(10), e14115. https://doi.org/10.2196/14115

Rangaprakash, D., Bohon, C., Lawrence, K. E., Moody, T., Morfini, F., Khalsa, S. S., Strober, M., & Feusner, J. D. (2018). Aberrant Dynamic Connectivity for Fear Processing in Anorexia Nervosa and Body Dysmorphic Disorder. *Frontiers in Psychiatry*, *9*, 273. https://doi.org/10.3389/fpsyt.2018.00273

Rubinov, M., & Sporns, O. (2010). Complex network measures of brain connectivity: Uses and interpretations. *Neuroimage*, *52*(3), 1059–1069.

Sanders, N., Smeets, P. A. M., Elburg, V., A, A., Danner, U. N., van Meer, F., Hoek, H. W., & Adan, R. A. H. (2015). Altered Food-Cue Processing in Chronically Ill and Recovered Women with Anorexia Nervosa. *Frontiers in Behavioral Neuroscience*, *9*. https://doi.org/10.3389/fnbeh.2015.00046

Spitzer, R. L., Kroenke, K., Williams, J. B., & Group, P. H. Q. P. C. S. (1999). Validation and utility of a self-report version of PRIME-MD: The PHQ primary care study. *Jama*, *282*(18), 1737–1744.

Spitzer, R. L., Kroenke, K., Williams, J. B., & Löwe, B. (2006). A brief measure for assessing generalized anxiety disorder: The GAD-7. *Archives of Internal Medicine*, *166*(10), 1092–1097.

Thompson, J. K., Cattarin, J., Fowler, B., & Fisher, E. (1995). The perception of teasing scale (POTS): A revision and extension of the physical appearance related teasing scale (PARTS). *Journal of Personality Assessment*, *65*(1), 146–157.

Townsend, J. T., & Ashby, F. G. (1983). *Stochastic modeling of elementary psychological processes*. CUP Archive.

Vuilleumier, P., & Pourtois, G. (2007). Distributed and interactive brain mechanisms during emotion face perception: Evidence from functional neuroimaging. *Neuropsychologia*, *45*(1), 174–194. https://doi.org/10.1016/j.neuropsychologia.2006.06.003

Welch, E., Birgegård, A., Parling, T., & Ghaderi, A. (2011). Eating disorder examination questionnaire and clinical impairment assessment questionnaire: General population and clinical norms for young adult women in Sweden. *Behaviour Research and Therapy*, *49*(2), 85–91.

Whiteside, S. P., & Lynam, D. R. (2001). The five factor model and impulsivity: Using a structural model of personality to understand impulsivity. *Personality and Individual Differences*, *30*(4), 669–689.

Whiteside, S. P., Lynam, D. R., Miller, J. D., & Reynolds, S. K. (2005). Validation of the UPPS impulsive behaviour scale: A four-factor model of impulsivity. *European Journal of Personality: Published for the European Association of Personality Psychology*, *19*(7), 559–574.

Wolff, M., Krönke, K.-M., Venz, J., Kräplin, A., Bühringer, G., Smolka, M. N., & Goschke, T. (2016). Action versus state orientation moderates the impact of executive functioning on real-life self-control. *Journal of Experimental Psychology: General*, *145*(12), 1635.

Wood, D. E., & Salzberg, S. L. (2014). Kraken: Ultrafast metagenomic sequence classification using exact alignments. *Genome Biology*, *15*(3), R46. https://doi.org/10.1186/gb-2014-15-3-r46
